# Partial response to anakinra in life-threatening Henoch-Schönlein purpura: case report

**DOI:** 10.1186/1546-0096-9-21

**Published:** 2011-08-11

**Authors:** Erynn M Boyer, Martin Turman, Kathleen M O'Neil

**Affiliations:** 1 Division of Rheumatology, Department of Pediatrics, University of Oklahoma Health Sciences Center, Oklahoma City, OK, USA; 2 Division of Nephrology Department of Pediatrics, University of Oklahoma Health Sciences Center, Oklahoma City, OK, USA

## Abstract

Henoch-Schönlein purpura is one of the most common forms of systemic vasculitis of childhood. We report the response to anakinra, the interleukin-1 receptor antagonist, in a 9 year old girl without prior medical problems who developed life-threatening Henoch-Schönlein vasculitis that produced renal failure, pulmonary hemorrhage and vasculitis of the brain. Her response supports the theory that interleukin-1 may be an important mediator in this disease. Further study of interleukin-1 antagonists in severe Henoch-Schönlein purpura may be warranted.

## Background

Henoch-Schönlein purpura (HSP) is the most common form of childhood vasculitis [1-8] affecting 10-20 per 100,000 children annually [1-3,5]. It occurs most frequently in children between the ages of 2 and 11 years [1]. HSP produces a broad array of clinical features resulting from widespread IgA deposition in the walls of small vessels [3]. Purpura of the skin, joint pain and swelling, gastrointestinal symptoms, and renal involvement are the most common manifestations with frequencies of 100% [2,3,9], 75% [2,3,8], 50-75%, and 40-50% [1-8] of the patient population, respectively. Less often, neurologic complications, pulmonary hemorrhage, and testicular vasculitis may occur [1-3]. Of these, glomerulonephritis remains the strongest indicator of poor prognosis [1,6,8]. Most series show that 1-7% of children with HSP glomerulonephritis will develop chronic renal failure [10-12]. Although reports describe the use of oral and parenteral corticosteroids, pulse methylprednisolone, dapsone, azathioprine, cyclophosphamide, cyclosporine, and plasmapheresis, researchers still lack strong evidence that confirms the superiority of any treatment regime in preventing tissue damage [4-6,13-15]. A recent metanalysis supports the use of glucocorticoids during acute disease, however [5].

Henoch-Schönlein purpura is typically a benign and self-limited systemic vasculitis. Perhaps because it does not frequently cause permanent damage, its pathogenesis remains relatively poorly understood. IgA deposition in vessel walls is a consistent pathologic finding, and is the hallmark of the disease [3]. The triggers that promote the IgA production and tissue deposition, those that determine why some individuals get the disease while most do not, and those that dictate why the disease can be life-threatening in a minority of affected children are not known.

A variety of therapeutic agents that target specific inflammatory cytokines are available for the treatment of rheumatic diseases. Biologic agents including soluble receptors and monoclonal antibodies that block the action of a variety of cytokines including tumor necrosis alpha, interleukin-1, interleukin-6 have been used with variable success in Kawasaki disease[16-19], granulomatosis with polyangiitis (Wegener granulomatosis) [20,21] and other severe forms of vasculitis [22-28]. Experimental evidence suggests inflammatory cytokines such as IL-1, TNF and IL-6 are involved in the pathogenesis of HSP [29]. Reports over the last 3 decades suggest that autoinflammatory diseases characterized by high interleukin-1 production such as familial Mediterranean fever may be associated with an increased incidence of HSP, and with more severe disease [7,30-39], making IL-1 an potential target in refractory disease.

Lead by data supporting an important role for IL-1 in HSP pathogenesis, we present a case that may indicate a direction for future research into novel therapies for severe Henoch-Schönlein purpura.

## Case Report

**Figure 1:**
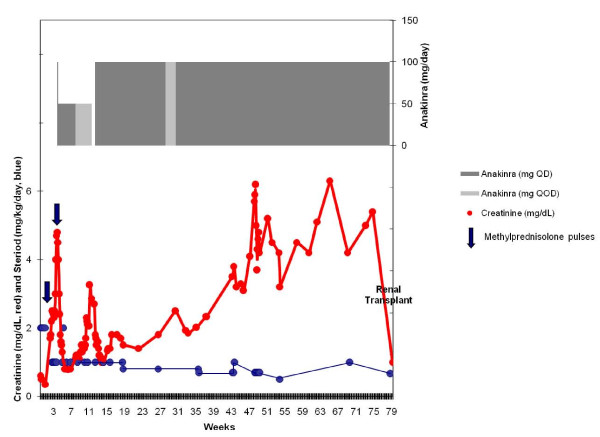
**Timeline of Response to Therapy**. Time in weeks after the onset of first symptoms of HSP is shown on the horizontal axis. The vertical axis shows serum creatinine in mg/dl (red), and therapeutic measures used. The daily dose of glucocorticoid in mg/kg/day is in blue; methylprednisolone pulse therapy is represented by blue arrows (30 mg/kg/day for 5 days, each arrow); and in the upper portion of the figure, anakinra dose is illustrated. Note that the serum creatinine rose when the anakinra dose was decreased, both at week eleven and at week thirty.

A 9 year old girl presented with a one week history of abdominal pain, vomiting, swelling and pain of the left ankle, dark colored urine and purpuric rash on her buttocks and lower extremities, consistent with a diagnosis of Henoch-Schönlein purpura nephritis. Her initial serum creatinine was normal at 0.5 mg/dL, but she had hypoalbuminemia (serum albumin was 2.7 mg/dL) and high grade proteinuria with urine protein:creatinine ratio of 15.1. After a three day course of 2 mg/kg/day methylprednisolone, her abdominal pain and rash improved. She was discharged on 2 mg/kg/day oral prednisone with plans to follow-up with her nephrologist in two weeks. Within a week, her HSP rash, abdominal pain and arthralgia returned, so she was admitted again to the hospital (see time course of her illness and treatment in Figure 1). She had nephrotic range proteinuria with a fall in serum albumin from 2.7 to 1.2 g/dL. Her serum creatinine had risen to 1.7 mg/dL, with blood urea nitrogen of 55 mg/dL. A kidney biopsy revealed crescents in more than half of the glomeruli, with sclerosing lesions and abundant IgA deposits indicative of severe HSPN. She received a three day course of 30 mg/kg/day methylprednisolone and began mycophenolate mofetil but failed to improve, with progressive rise in serum creatinine over four days to 2.5 mg/dL.

**Figure 2:**
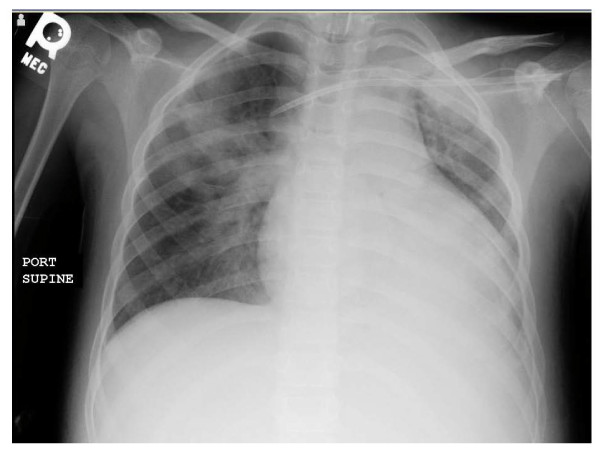
**Chest X-ray showing pulmonary hemorrhage**. Diffuse alveolar opacification is apparent, particularly in the right upper lobe. Bronchoalveolar lavage revealed fresh blood and hemosiderin laden macrophages.

**Figure 3:**
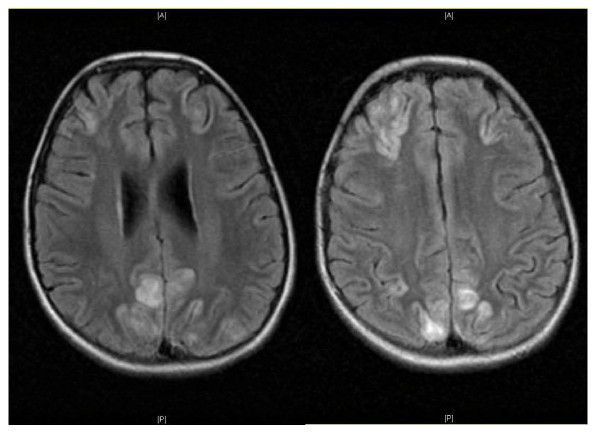
**Brain Magnetic Resonance Imaging**. These two coronal FLAIR images demonstrate increased signal intensity within the cortical gray matter of the parietal, occipital and frontal lobes, with minimal involvement of the sub-cortical white matter. The deep white matter is spared. This picture is consistent with scattered regions of vasogenic edema most consistent with vasculitis, and not indicative of posterior reversible encephalopathy from hypertension.

At the time she was readmitted, she had mild dyspnea and hypoxemia. Despite vigorous diuresis and broad spectrum antimicrobial coverage, her condition progressed to respiratory failure by the second week. Chest radiography showed diffuse patchy infiltrates (Figure 2). Bronchoalveolar lavage confirmed pulmonary hemorrhage grossly, with fresh blood and hemosiderin laden macrophages identified in the lavage fluid. Because of the child's rising creatinine and lack of response to more conventional treatments, she received IVIG therapy and a 2 week course of plasmapheresis, but had little clinical response. After a week of plasmapheresis she developed initially focal, then generalized seizures and required anticonvulsant therapy. She had altered mental status with sluggish cognition. MRI of the brain showed numerous regions of vasogenic edema consistent with cerebral vasculitis (Figure 3). The pattern of edema was not suggestive of posterior reversible encephalopathy syndrome secondary to hypertension. By this time, the serum creatinine was 4.8 mg/dL.

Based on evidence that severe HSP may be characterized by interleukin-1 (IL-1) over-production [7,34,40], a planned one-day trial of anakinra (recombinant IL-1 receptor antagonist) was initiated in the hopes of avoiding the toxicity of cyclophosphamide therapy. Accordingly, her renal function rapidly improved and serum creatinine fell from 4.8 mg/d to 2.4 in 48 hours, and further to 1.0 in 10 days. Both neurologic and pulmonary dysfunction cleared rapidly, and she was weaned from ventilator within 24 hours. Anakinra was continued daily for 6 weeks, and then it was discontinued, because the child objected strongly to the injections, her serum creatinine was stable at 0.8 mg/dL, and it was unclear whether anakinra was the cause of her improvement.

Within one week of stopping the anakinra, the serum creatinine rose to 3.26 mg/dL despite continuous prednisone (1 mg/kg/day) and mycophenolate mofetil. Renal biopsy at this time again showed active vasculitis with evidence of progression with early interstitial fibrosis and tubular atrophy, as well as progression from cellular to fibrous crescents, and global glomerulosclersosis of 20% of glomeruli. Because of the rising creatinine and biopsy findings, she resumed daily anakinra at twice her previous dose (100 mg/day) and received another course of plasmapheresis. She had persistent hypertension during this period requiring a variety of medications. After resuming anakinra, her renal function and hypertension improved and remained stable with serum creatinine between 1.0 and 1.8 mg/dL (higher levels with lower prednisone doses).

Four months after resuming anakinra, an attempt was made to wean the injections to every other day. The creatinine rose from 1.8 to 2.5 mg/dL within two weeks, so she returned to daily dosing (100 mg/day). Unfortunately, her renal function improved only transiently, and her creatinine again rose. Her glomerulonephritis was unresponsive to higher doses of glucocorticoid, and renal failure ensued. Eleven months after her initial diagnosis, a renal biopsy was performed to determine whether further aggressive immunosuppression was warranted. This biopsy revealed global glomerulosclerosis involving nearly 100% of the glomeruli and marked interstitial fibrosis with tubular atrophy. She began dialysis two weeks later. Fourteen months after her initial diagnosis, she received a successful renal transplant.

## Discussion

In the child described in this report with life-threatening systemic vasculitis that caused renal failure, pulmonary hemorrhage and diffuse vasculitis of the brain with seizures and impaired cognition, there was no prior medical history to suggest an underlying autoinflammatory condition characterized by over-production of interleukin-1. In fact, subsequent DNA analysis was negative for the common familial Mediterranean fever gene mutations. The severe HSP complications occurred despite plasmapheresis, pulse methylprednisolone and high dose mycophenolate mofetil. In an attempt to avoid the toxicity of cyclophosphamide [41-43], a one-day trial of subcutaneous anakinra was undertaken with rapid and dramatic improvement. The HSP relapsed twice when the anakinra was tapered, and she improved with reinstitution of the drug. Unfortunately, the disease ultimately progressed to irreversible renal failure. Thus in retrospect, either the dose of anakinra should not have been decreased, or cyclophosphamide might have proved a wiser choice of treatment.

## Conclusions

Although this single case report does not provide sufficient evidence to indicate that treatment with an interleukin-1 antagonist is either effective or safe in severe HSP, it does suggest that disease may be ameliorated with anakinra, and moreover, that further study of the role of interleukin-1 in HSP and of IL-1 antagonism in severe disease might be appropriate.

## Consent

The parent of this minor child has given written informed consent for the publication of the child's case report and accompanying de-identified images. A copy of the written consent is available for review by the Editor-in-Chief of this journal.

## List of abbreviations

DNA: deoxyribonucleic acid; FMF: familial Mediterranean fever; HSP: Henoch-Schönlein purpura; IgA: immunoglobulin A; IL-1: interleukin-1; IL-1RA: interleukin-1 receptor antagonist; IVIG: intravenous immunoglobulin; MEFV: gene encoding pyrin, mutated in familial Mediterranean fever; MR: magnetic resonance; MRI: magnetic resonance imaging.

## Competing interests

The authors declare that they have no competing interests.

## Authors' contributions

EMB reviewed the medical records of this child, drafted the manuscript and designed the figure. MT was the nephrologist managing the patient throughout her course, and contributed to writing the manuscript. KMO was the rheumatologist consulting on this patient, and wrote the literature review and background of the manuscript. All authors read and approved the final manuscript.
